# The effects of fossil taxa, hypothetical predicted ancestors, and a molecular scaffold on pseudoextinction analyses of extant placental orders

**DOI:** 10.1371/journal.pone.0257338

**Published:** 2021-09-17

**Authors:** Peggy L. Brady, Mark S. Springer

**Affiliations:** Department of Evolution, Ecology, and Evolutionary Biology, University of California, Riverside, Riverside, CA, United States of America; Indiana University Bloomington, UNITED STATES

## Abstract

Pseudoextinction analyses, which simulate extinction in extant taxa, use molecular phylogenetics to assess the accuracy of morphological phylogenetics. Previous pseudoextinction analyses have shown a failure of morphological phylogenetics to place some individual placental orders in the correct superordinal clade. Recent work suggests that the inclusion of hypothetical ancestors of extant placental clades, estimated by ancestral state reconstructions of morphological characters, may increase the accuracy of morphological phylogenetic analyses. However, these studies reconstructed direct hypothetical ancestors for each extant taxon based on a well-corroborated molecular phylogeny, which is not possible for extinct taxa that lack molecular data. It remains to be determined if pseudoextinct taxa, and by proxy extinct taxa, can be accurately placed when their immediate hypothetical ancestors are unknown. To investigate this, we employed molecular scaffolds with the largest available morphological data set for placental mammals. Each placental order was sequentially treated as pseudoextinct by exempting it from the molecular scaffold and recoding soft morphological characters as missing for all its constituent species. For each pseudoextinct data set, we omitted the pseudoextinct taxon and performed a parsimony ancestral state reconstruction to obtain hypothetical predicted ancestors. Each pseudoextinct order was then evaluated in seven parsimony analyses that employed combinations of fossil taxa, hypothetical predicted ancestors, and a molecular scaffold. In treatments that included fossils, hypothetical predicted ancestors, and a molecular scaffold, only 8 of 19 pseudoextinct placental orders (42%) retained the same interordinal placement as on the molecular scaffold. In treatments that included hypothetical predicted ancestors but not fossils or a scaffold, only four placental orders (21%) were recovered in positions that are congruent with the scaffold. These results indicate that hypothetical predicted ancestors do not increase the accuracy of pseudoextinct taxon placement when the immediate hypothetical ancestor of the taxon is unknown. Hypothetical predicted ancestors are not a panacea for morphological phylogenetics.

## Introduction

Charles Darwin [1] provided the world with its first glimpse of a modern phylogeny with contemporary taxa deployed at its tips and ancestors at internal nodes. Ever since, evolutionary biologists have attempted to decipher the relationships between living and extinct members of the Tree of Life. Morphological characters were the main source of data for phylogenetic analyses for much of the 20^th^ century [2, 3], but since the 1960s systematists have increasingly relied on different types of genetic data and now genomic data with the advent of massively parallel DNA sequencing [3, 4]. Molecular data are preferred by most systematists due to large sample size [2, 3, 5] and greater phylogenetic reliability [6]. Whereas molecular data are readily available for many extant taxa, the procurement of DNA sequences has proven much more challenging for recently extinct species and impossible for long extinct species [7]. The dearth of molecular data for most extinct species, which comprise the vast majority of species that have lived on Earth, mandates the continued use of morphological data to study the phylogenetic relationships of extinct taxa to each other and to extant taxa [5].

Despite this demonstrated need, morphological characters are now rarely collected for phylogenetic studies of extant taxa [8]. Thus, many contemporary phylogenies are built using only molecular data, although fossils remain critical for time-scaling these phylogenies [3]. Additionally, the inclusion of fossil taxa in phylogenetic analyses can be critical for elucidating temporal sequences of character evolution because extinct taxa may preserve morphological character combinations that are not present in extant taxa [9, 10]. To include extinct species in phylogenetic analyses we need to continue to collect morphological data [8, 11]. At the same time, we must also assess the accuracy of morphological phylogenetic methods given potential problems with correlated homoplasy that can result in morphological support for groups that are polyphyletic based on molecular data [12–15].

In the case of the 19 extant orders of placental mammals, there are both congruent and incongruent clades that have emerged from molecular and morphological studies of interordinal relationships. Both types of data provide robust support for Glires and Paenungulata, but there are far more cases of incongruence [13]. Molecular data sets provide incontrovertible support for four superorders of placental mammals: Afrotheria, Xenarthra, Laurasiatheria, and Euarchontoglires [16–21]. However, only Xenarthra has been recovered by traditional morphological data sets [12]. Instead, morphological phylogenies have recovered groups such as Edentata (Xenarthra + Pholidota), Lipotyphla, Ungulata, and Volitantia [22–26], all of which are incompatible with one or more of the four molecular superorders. These problems with the phylogenetic placement of extant placental orders based on morphological phylogenetics have raised concerns pertaining to the reliability of morphology to place extinct taxa in the eutherian tree [12, 15, 27].

Previous studies have examined the placement of extinct taxa in phylogenies by simulating extinction in extant taxa (also called ‘pseudoextinction’) for which phylogenetic relationships are robustly supported. This is achieved by coding molecular characters and soft tissue characters as missing for each pseudoextinct taxon prior to phylogenetic analysis [12, 15, 28–30]. These techniques have been applied to species within Tenrecidae [28], placental mammal orders [12, 15], groups of vertebrates and invertebrates [30], and species within the order Primates [29]. Asher and Hofreiter [28] and Pattinson et al. [29] concluded that morphological data sets were reliable for phylogenetic reconstruction whereas Springer et al. [12, 15] and Sansom & Wills [30] found that morphological data sets were inadequate for reconstructing the relationships of pseudoextinct taxa. While morphology may be able to place extinct species within extant families or orders [28, 29], the accuracy of morphology for placing more inclusive pseudoextinct groups is poor [12, 15, 30]. Thus, morphology appears to reconstruct fossil relationships more accurately when a pseudoextinct species has an extant, close relative that can be placed using molecular data.

Small sample size was noted as one potential reason for the poor performance of morphology in reconstructing higher-level relationships among placental mammals [12]. All but one of the aforementioned pseudoextinction studies [15] were based on an average of <500 morphological characters for each pseudoextinct group.

The large data set of O’Leary et al. [31], which contains >4500 characters, allowed pseudoextinction analyses to be revisited with a significantly increased morphological sample size [15]. Thirteen of the 18 placental orders (72%) moved to an interordinal position that violated the well-supported molecular scaffold when they were treated as pseudoextinct (Xenarthra [Cingulata + Pilosa] was treated as a single order in [15]). Three of these orders (Afroscoricida, Eulipotyphla, Cetartiodactyla) were also rendered polyphyletic. Only Lagomorpha, Rodentia, Proboscidea, Sirenia, and Hyracoidea retained their correct interordinal positions [15]. These results showcase that even large morphological data sets are subject to the problems of homoplasy.

Beck and Baillie [32] assessed the accuracy of morphological phylogenetics when hypothetical predicted ancestors (HPAs) were added to O’Leary et al.’s [31] morphological data set. These authors enforced a molecular scaffold from Meredith et al. [33] for 46 extant mammals and reconstructed ancestral states for each internal node. All of these predicted ancestors were then added to O’Leary et al.’s [31] morphological matrix and their impact on phylogeny reconstruction was assessed in unconstrained phylogenetic analyses. Beck and Baillie [32] recovered a morphological phylogeny that is largely congruent with Meredith et al.’s [33] DNA-based phylogeny including the correct placement of each placental order in its well-supported superorder (i.e., Afrotheria, Xenarthra, Euarchontoglires, Laurasiatheria). An important conclusion from this study is that predicted ancestors have the potential to reconcile estimates of phylogeny that are inferred from morphological and molecular data sets. Indeed, application of Beck and Baillie’s [32] method resulted in most of the same clades that were present in the molecular scaffold that was used to reconstruct ancestral morphological characters. Similar results were obtained by Asher et al. [34] when these methods were applied to a 219-character morphological data set for Glires.

It is important to note that Beck and Baillie [32] and Asher et al. [34] used hypothetical predicted ancestors for different purposes. Beck and Baillie’s [32] analyses with hypothetical predicted ancestors represented an ideal scenario for finding real fossils with character combinations that can link morphologically disparate mammalian taxa without the need for molecular data. As noted by these authors, if "genuine fossil taxa exhibit these character combinations, and are sufficiently well preserved, then their discovery and inclusion in phylogenetic analyses might be sufficient to largely resolve the current conflict between molecular and morphological analyses…" Asher et al. [34] viewed Beck and Baillie’s [32] method as a novel approach to incorporate the phylogenomic signal from modern taxa into a phylogenetic analysis without "having to sample non-fossilizable data, such as DNA." However, it remains unclear if the inclusion of predicted ancestors for extant taxa will result in more accurate phylogenies for extinct taxa for which direct ancestors cannot be predicted with Beck and Baillie’s [32] method. This is because most extinct taxa lack molecular data and cannot be included as tips on a molecular phylogeny that is used to reconstruct ancestral states.

Here we address this issue with O’Leary et al.’s [31] large morphological data set. To do this, we combined pseudoextinction [12, 28] and HPAs [32, 34] to assess if inclusion of the latter results in the accurate placement of pseudoextinct taxa for which the immediate ancestor cannot be reconstructed. We also examine the impact of including different combinations of molecular scaffolds, predicted ancestors, and real fossils on recovering accurate phylogenies for pseudoextinct taxa.

## Methods

### Morphological data set

Morphological data were obtained from Morphobank project 773 [31]. The unaltered data set contained a total of 4,541 unordered morphological characters that were scored across 86 mammalian taxa, 40 of which are extinct. Specifically, we employed the data set that was used for maximum likelihood analyses in RAxML by O’Leary et al. [31]. This matrix differs from the original matrix by containing no letters for any coded morphological character. This means that the total number of possible character states for any given character was 10 (0–9) instead of 19 (0–9 and: E, F, I, J, L, O, P, Q, X). O’Leary et al. [29] recoded character states that were above 9 as missing (?) while any character states coded as 0–9 were left intact. This change affected twelve morphological characters. This matrix also differs from the original matrix in that all polymorphic character scorings were recoded as missing (?). These changes increased the number of constant characters from 403 to 407.

Each extant order of placental mammals was represented by at least one taxon. Nine of these orders were represented by two or more species. For several of these analyses, the extinct taxa were removed from the matrix, leaving only the 46 extant taxa. Forty-two of these are placentals, two are marsupials, and two are monotremes. Following Beck and Baillie [32] we updated *Thomashuxleya* with morphological characters from a more recent study [35] and removed 407 characters that were constant across all 86 taxa.

### Molecular scaffold

**Fig 1:**
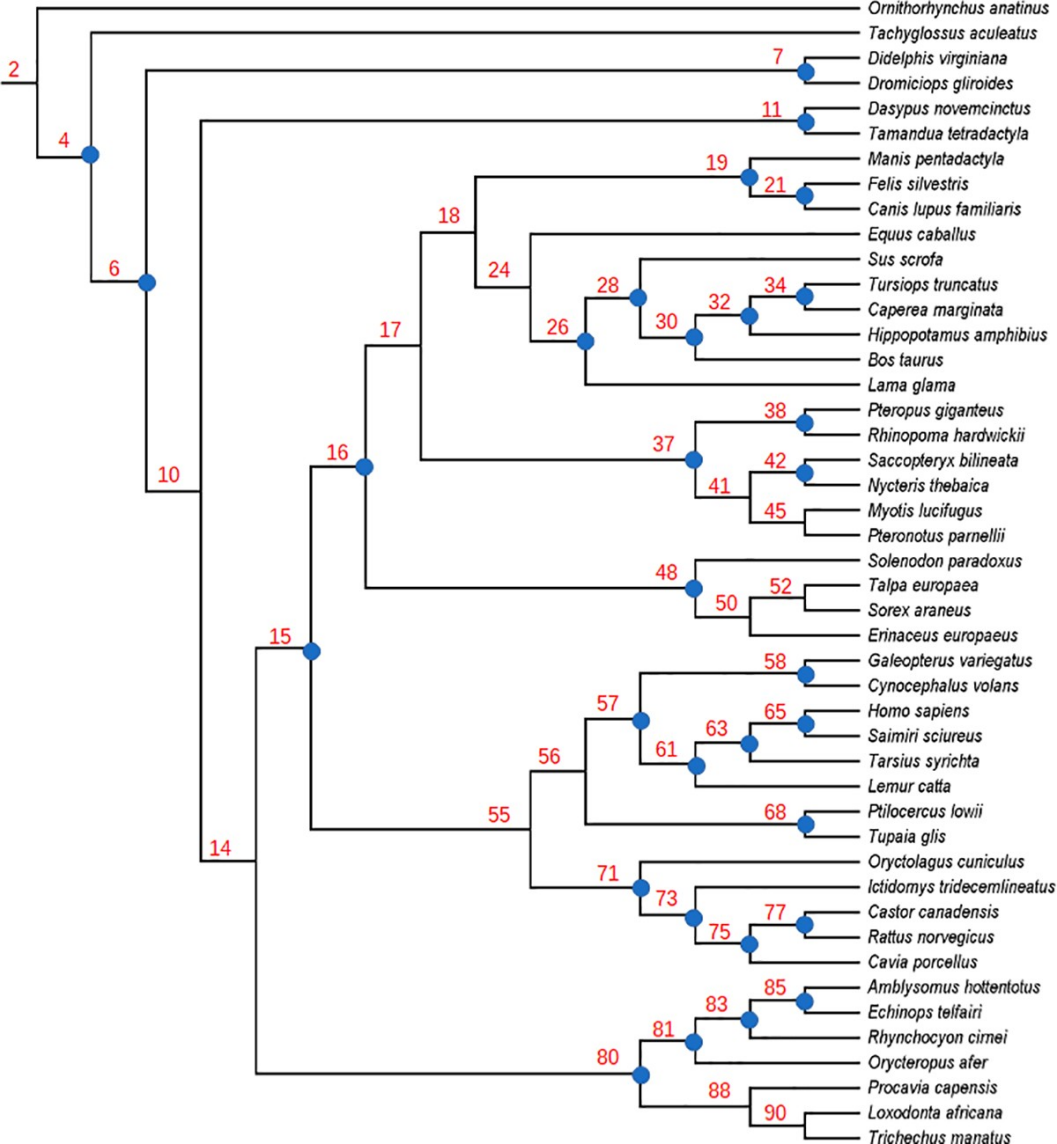
Molecular scaffold species tree. Our fully bifurcating molecular scaffold species tree (20588 steps) that was used for ancestral state reconstructions. The tree was rooted with the monotreme *Ornithorhynchus anatinus* (platypus). Each internal node has a designated number in red. This number corresponds with the number assigned to each hypothetical predicted ancestor (HPA) in our matrices. These matrices are available in figshare (10.6084/m9.figshare.15183258). Internal nodes that were constrained in the molecular scaffold are denoted with blue circles.

We employed a well-supported molecular scaffold [36] based on Meredith et al.’s [33] phylogenetic analyses of 33,030 base pairs (bp) of protein coding DNA (21 gene segments) and 2,573 bp of DNA from untranslated regions (five gene segments). Clades that were included in our molecular scaffold are also well supported by other molecular studies [16, 37–40]. Our molecular scaffold is illustrated in Fig 1 and included all 46 extant taxa in O’Leary et al.’s [31] data set. This scaffold is fully bifurcating with the exception of the following polytomies: 1. ((Dermoptera + Primates), Scandentia, Glires); 2. (*Talpa*, *Erinaceus*, *Sorex*); 3. ((*Saccopteryx + Nycteris*), *Myotis*, *Pteronotus*); 4. ((Pholidota + Carnivora), Perissodactyla, Cetartiodactyla, Chiroptera); 5. (Hyracoidea, Proboscidea, Sirenia); and 6. (Xenarthra, Afrotheria, Boreoeutheria). We retained these polytomies in all of our scaffold analyses because these nodes were resolved differently in DNA versus amino acid analyses or were not strongly supported (bootstrap support < 90%; Bayesian posterior probabilities < 0.95) [33]. The morphological data of the extant taxa from O’Leary et al. [31] was analyzed with parsimony (see below) and our molecular scaffold to obtain a fully bifurcating molecular scaffold species tree (MSST) (see Fig 1) that was used for ancestral state reconstructions and tree-to-tree comparisons with Robinson and Foulds [41] distances (see below). The molecular scaffold was also employed as a backbone constraint (i.e., containing only a subset of the included taxa) in analyses that included extinct taxa. Finally, extant taxa for each extant placental order were dropped from the molecular scaffold in analyses that treated these orders as pseudoextinct.

### Pseudoextinct matrices

O’Leary et al.’s [31] phenomic matrix includes both osteological (1–3,660) and non-osteological (3,661–4,541; total of 881) characters. The latter category includes both soft tissue and behavioral characters. For pseudoextinct taxa, only osteological characters were retained for parsimony analyses. Non-osteological characters were scored as missing (?) for pseudoextinct taxa to mimic the usual absence of these characters in extinct taxa that are only known from fossilized remains. Matrix alterations were performed in Mesquite [42]. After the removal of the 407 constant characters, 4,134 characters remained (3,371 of which were osteological).

### Ancestral state reconstructions

The MSST constructed with O’Leary et al.’s [31] morphological data set and our molecular scaffold was loaded into Mesquite. Ancestral state reconstructions (ASR) for each node on our fully bifurcating species tree (Fig 1) were performed with parsimony using the ‘Trace all characters’ command. This procedure was replicated for each pseudoextinct data set, i.e., the order in question was removed from the MSST prior to parsimony ancestral state reconstruction, and ASRs were constructed for a tree that did not include the pseudoextinct order. These reconstructions were exported into Excel where all ambiguously reconstructed characters (i.e., with two or more possible states) were removed and replaced with a ‘?’. Following these alterations, each reconstructed data set was run through the same R script used by Beck and Baillie [32] to ensure that the hypothetical reconstructed ancestors were not coded for any characters that are inapplicable given the reconstructed state at another character. We did not alter this script except to change the name of the input (line 49) and output (line 85) file names. This code is available in figshare (10.6084/m9.figshare.15183372).

We did not explore maximum likelihood ASRs because Beck and Baillie [32] found that the two optimality criterion used in their study (maximum parsimony and maximum likelihood) both fit the Meredith et al. [33] topology equally well when measured by Robinson-Foulds distances [41]. Additionally, in analyses that included all of the hypothetical predicted ancestors, maximum parsimony analyses were more accurate than maximum likelihood analyses in reconstructing monophyletic superordinal clades.

### Phylogenetic reconstructions

Each matrix was analyzed in TNT [43] using a traditional search and TBR branch swapping. *Morganucodon oehleri* was used as the outgroup for all analyses that included fossil taxa. *Ornithorhynchus anatinus* was used as the outgroup in analyses that omitted fossil taxa. We used maximum parsimony with stepwise addition and 10,000 random taxon addition sequences to search for the shortest tree in each analysis. We did not perform maximum likelihood analyses because there is evidence that parsimony performs better than other optimality criterion for morphological phylogenetic analyses [44–46]. Furthermore, there are concerns about the appropriateness of likelihood models for morphological data since many of these models were designed to fit molecular data [3, 47, 48]. Seven different sets of analyses ((Scaffold + Fossils + HPA); (Scaffold + Fossils); (Scaffold + HPA); (Fossils + HPA); Scaffold; Fossils; HPA) were conducted using different combinations of fossil taxa, HPAs, and our molecular scaffold. In each analysis, all extant taxa were included. Trees were saved as unrooted. For analyses that were performed with the combination of a molecular scaffold + HPAs, we set the maximum number of saved trees in TNT to 100,000 (10,000 random addition sequences x 10 trees per addition sequence). With the exception of Eulipotyphla (99,520 trees), all of the analyses with a molecular scaffold + HPAs resulted in the maximum number of saved trees. The large number of saved trees in the molecular scaffold + HPAs analysis resulted from each HPA having between one and three most parsimonious placements. For example, the HPA for node 65 (Fig 1) between *Homo sapiens* and *Saimiri sciureus* has two most parsimonious placements, as a sister taxon to either *S*. *sciureus* or (*H*. *sapiens* + *S*. *sciureus*). However, after pruning all HPAs from these Scaffold + HPA trees, all trees for each pseudoextinct order condensed to a single topology for the 46 extant taxa.

### Tree comparisons

Following our analyses, all trees were exported in NEXUS format and imported into PAUP 4.0a [49]. Once in PAUP, any HPAs and/or fossil taxa were pruned from the tree and any duplicate topologies that resulted from this pruning were discarded. All remaining most parsimonious trees, each of which included 46 extant taxa, were then compared to the MSST using Robinson-Foulds (RF) distances [41]. RF distances measure the number of splits (internal branches) that are unique to one of the two trees. All tree comparisons were based on unrooted trees.

## Results

**Fig 2:**
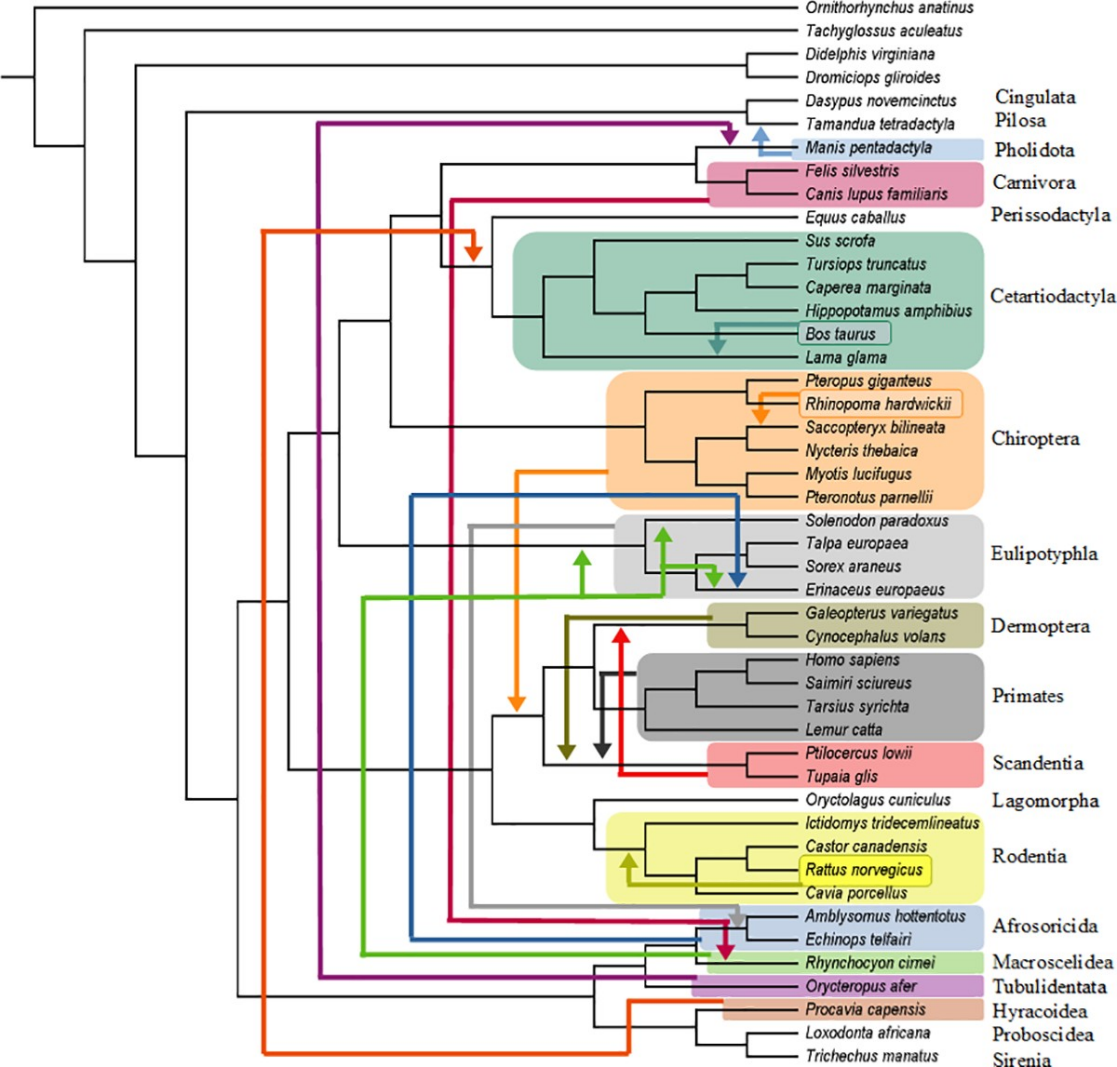
Summary of molecular scaffold + hypothetical predicted ancestors + fossil taxa analyses. Summary of the pseudoextinction results for the O’Leary et al. [31] data set for one of our seven sets of analyses (molecular scaffold + hypothetical predicted ancestors + fossil taxa). Orders that were recovered in phylogenetic positions that conflict with the molecular scaffold are indicated by arrows. All analyses recovered a single most parsimonious tree except for Macroscelidea, which had three most parsimonious reconstructions. Shifts of individual species within orders are also indicated by arrows. Only eight orders (42%) (Proboscidea, Sirenia, Lagomorpha, Perissodactyla, Cingulata, Pilosa, Rodentia, Cetartiodactyla) remained in the correct interodinal position when pseudoextinct.

**Fig 3:**
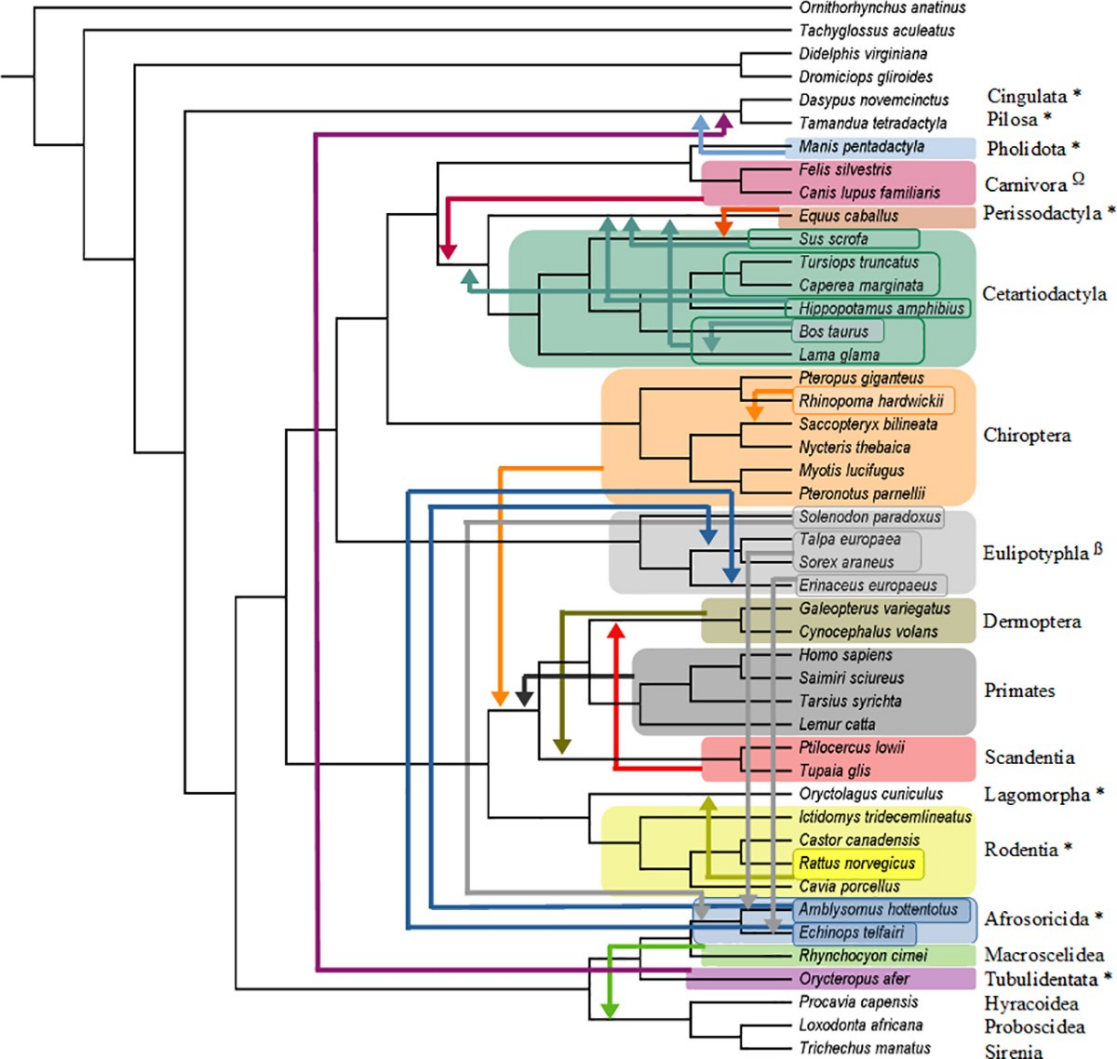
Summary of molecular scaffold + fossil taxa analyses. Summary of the pseudoextinction results for the O’Leary et al. [31] data set for one of our seven sets of analyses (molecular scaffold + fossil taxa). Orders that were recovered in phylogenetic positions that conflict with the molecular scaffold are indicated by arrows. Any shifts of individual species are also indicated by arrows. All analyses found a single most parsimonious tree (MPT) except for Eulipotyphla and Sirenia, each of which had two most parsimonious reconstructions. One MPT for Eulipotyphla supported Epitheria (Afrotheria + Boreoeutheria), as shown above, while the second MPT supported Exafroplacentalia (Xenarthra + Boreoeutheria). One MPT for Sirenia supported Epitheria (Afrotheria + Boreoeutheria), as shown above, while the second MPT supported Atlantogenata (Xenarthra + Afrotheria). In both cases the relationships for the pseudoextinct Eulipotyphla and Sirenia were unchanged between their two MPTs. Only six orders (32%) (Proboscidea, Sirenia, Lagomorpha, Hyracoidea, Cingulata, Pilosa) remained in the correct interordinal position when pseudoextinct. * = MPT supports Atlantogenata, ß = MPT supports Chiroptera as sister to Ferae (Carnivora + Pholidota), Ω = MPT supports Chiroptera as sister to Pholidota.

**Fig 4:**
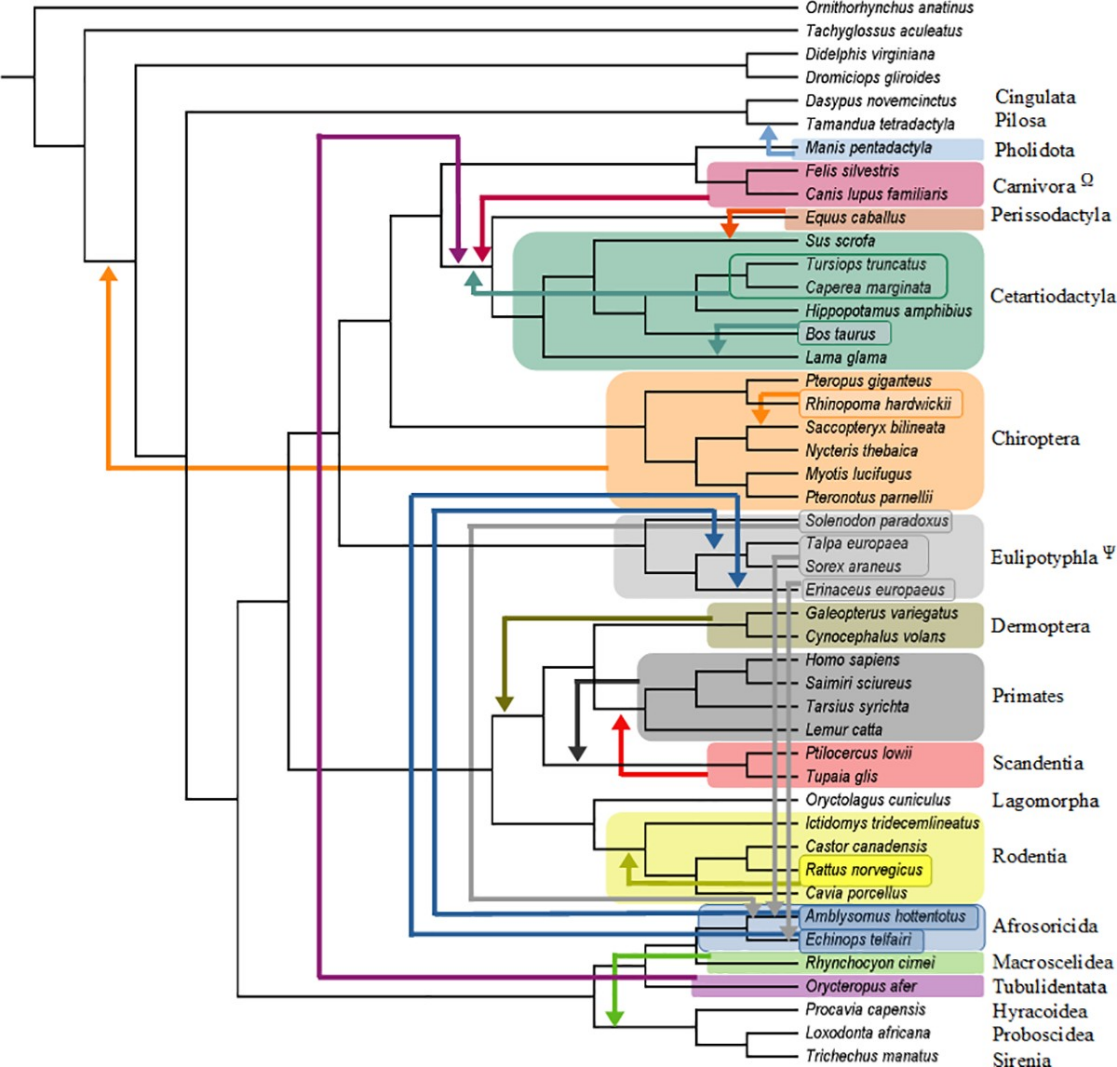
Summary of molecular scaffold analyses. Summary of the pseudoextinction results for the O’Leary et al. [31] data set for one of our seven sets of analyses (molecular scaffold only). Orders that were recovered in phylogenetic positions that conflict with the molecular scaffold are indicated by arrows. Any shifts of individual species are also indicated by arrows. All analyses recovered a single most parsimonious tree (MPT). Only seven orders (37%) (Proboscidea, Sirenia, Lagomorpha, Hyracoidea, Cingulata, Pilosa, Rodentia) remained in the correct interordinal position when pseudoextinct. Ψ = MPT supports Chiroptera as sister to (Perissodacyla + Cetartiodactyla), Ω = MPT supports Chiroptera as sister to Pholidota.

**Fig 5:**
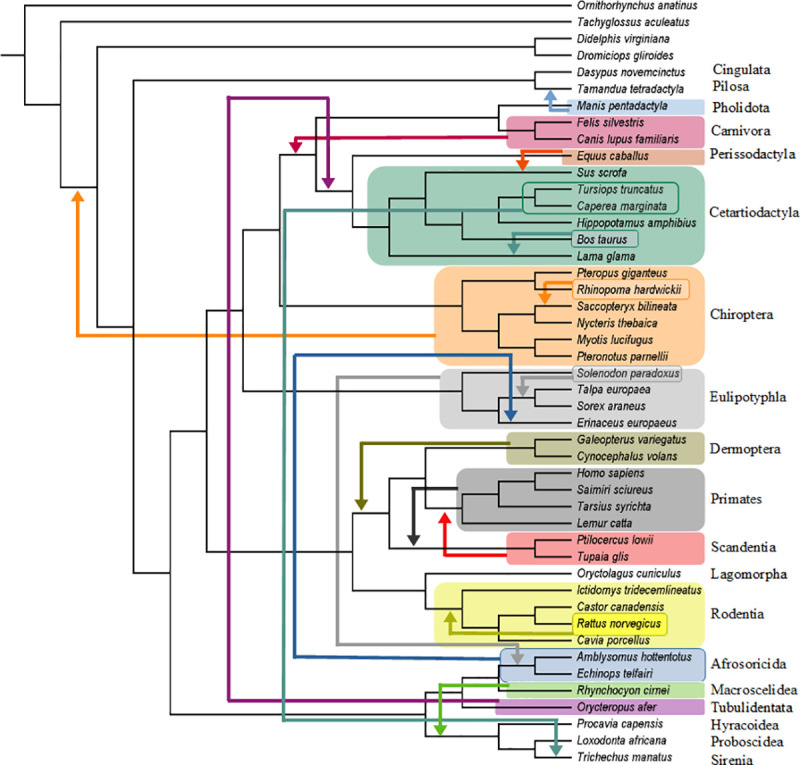
Summary of molecular scaffold + hypothetical predicted ancestors analyses. Summary of the pseudoextinction results for the O’Leary et al. [31] data set for one of our seven sets of analyses (molecular scaffold + hypothetical predicted ancestors). Orders that were recovered in phylogenetic positions that conflict with the molecular scaffold are indicated by arrows. Any shifts of individual species are also indicated by arrows. All analyses recovered a single most parsimonious tree (MPT). Only seven orders (37%) (Proboscidea, Sirenia, Lagomorpha, Hyracoidea, Cingulata, Pilosa, Rodentia) remained in the correct interordinal position when pseudoextinct.

Phylogenetic trees based on seven different analyses with O’Leary et al.’s [31] morphological data set were compared to our MSST using RF distances. The results of these comparisons are summarized in Table 1. Table 2 summarizes the misplacement of pseudoextinct taxa relative to the full molecular scaffold for all seven analyses. The results of these analyses are also summarized in Figs 2–5 and S1–S3 Figs. Phylogenetic trees with pseudoextinct taxa, before and after pruning fossils and/or HPAs, are provided in Newick format in figshare (10.6084/m9.figshare.15183294 and 10.6084/m9.figshare.15183345).

**Table 1:** Summary of Robinson-Foulds comparisons.

		RF distance to MSST						
Pseudoextinct order	Scaffold + Fossils + HPAs	Fossils + HPAs	Scaffold + Fossils	Scaffold + HPAs	Fossils	HPAs	Scaffold	Row Mean/Median
Afrosoricida	14	32	18	14	44	40	16	25.43/18
Carnivora	16	32,34	4	2	44,46	32	4	19.43/16
Cetartiodactyla	4	30	8	24	44	42,42	8	22.86/24
Chiroptera	12	36	12	16	44	40	16	25.14/16
Cingulata	0	32	2	0	44	36	0	16.29/2
Dermoptera	2	18	2	2	44	34	2	14.86/2
Eulipotyphla	10	28,28	18,20	14	44	38	18	24.43/19
Hyracoidea	12	34	0	0	44,46	32	0	17.57/12
Lagomorpha	0	20	2	0	46	36	0	14.86/2
Macroscelidea	12,10,14	36,34	4	4	44	34	4	19.57/12
Perissodactyla	0	18	6	4	44	36	4	16/6
Pholidota	14	32	12	14	44	34	14	23.43/14
Pilosa	0	32	2	0	44	34	0	16/2
Primates	2	20	2	2	44	34	2	15.14/2
Proboscidea	0	30	0	0	48	34,34,34	0	16/0
Rodentia	4	22	8	4	46	46,44	4	19/8
Scandentia	2	18,20	2	2	44	38	2	15.57/2
Sirenia	0	20	0,2	0	44	34,34,34	0	14.14/1
Tubulidentata	14	32,30,32	6	12	44	34,34	12	21.9/14
Column Mean/ Median	6.21/4	27.49/30	5.79/4	6/2	44.53/44	36.16/34	5.58/4	

Results of Robinson-Foulds (RF) comparisons for phylogenetic analyses with a pseudoextinct order versus the molecular scaffold species tree. When multiple most parsimonious trees were found, all RF distances are listed. Abbreviations: HPA, hypothetical predicted ancestors.

**Table 2:** Summary of misplacements of pseudoextinct taxa relative to the molecular scaffold.

Pseudoextinct Order	Scaffold + Fossils + HPAs	Fossils + HPAs	Scaffold + Fossils	Scaffold + HPAs	Fossils	HPAs	Scaffold	Row Totals (# of Occurrences)
Afrosoricida	1,2	1,2	1,2,4	1,2	1,2,4	1,2	1,2,4	1: 7X; 2: 7X; 4: 3X
Carnivora	1,2	1,2	1	1	1,2	1,2	1	1: 7X; 2: 4X
Cetartiodactyla	5	2,5	4	1,2,4	2,5	1,2,4	4	1:2X; 2: 4X; 4: 4X; 5: 3X
Chiroptera	1,2,5	1,2,5	1,2,5	1,2,5	1,2,5	1,2,5	1,2,5	1: 7X; 2: 7X; 5: 7X
Cingulata		3			3	1,2		1: 1X; 2: 1X; 3: 2X
Dermoptera	1	1	1	1	1,2	1,3	1	1: 7X; 2:1X; 3: 1X
Eulipotyphla	1,2	1,2	1,2,4	1,2,5	1,2,4	1,2,5	1,2,4	1: 7X; 2: 7X; 4: 3X; 5: 2X
Hyracoidea	1,2	1,2			1,2	3		1: 3X; 2: 3X; 3: 1X
Lagomorpha		1			2	1,3		1: 2X; 2: 1X; 3: 1X
Macroscelidea	1,2	1,2	1	1	1,2	1,3	1	1: 7X; 2: 3X; 3: 1X
Perissodactyla			1	1	2	1,2	1	1: 4X; 2: 2X
Pholidota	1,2	1,2	1,2	1,2	1,2	1,2	1,2	1: 7X; 2: 7X
Pilosa		1,2			3	1,2		1: 2X; 2: 2X 3: 1X
Primates	1	1	1	1	1,2	1,3	1	1: 7X; 2: 1X; 3: 1X
Proboscidea		2			2	3		2: 2X; 3: 1X
Rodentia	5	2,5	1,4	5	1,2,4	2,5	5	1: 2X; 2: 3X; 4: 2X; 5: 5X
Scandentia	1	1	1	1	1,2	1,3	1	1: 7X; 2: 1X; 3: 1X
Sirenia					2	3		2: 1X; 3: 1X
Tubulidentata	1,2	1,2	1,2	1,2	1,2	1,2	1,2	1: 7X; 2: 7X
	1: 11X	1: 13X	1: 12X	1: 12X	1: 12X	1: 15X	1: 11X	
Column Totals (# of Occurrences)	2: 8X	2: 12X	2: 5X	2: 6X	2: 17X	2: 11X	2: 5X	
	5: 3X	3: 1X	4: 4X	4: 1X	3: 2X	3: 8X	4: 3X	
		5: 3X	5: 1X	5: 3X	4: 3X	4: 1X	5: 2X	
					5: 2X	5: 3X		

Summary of misplacement of pseudoextinct taxa relative to the full molecular scaffold in seven different phylogenetic analyses. 1 = pseudoextinct taxon shows incorrect sister-group relationship; 2 = correct superordinal group (Afrotheria, Euarchontoglires, Laurasiatheria, Xenarthra) for pseudoextinct taxon is not monophyletic; 3 = correct superordinal group for pseudoextinct order is monophyletic, but other superordinal group(s) are incorrect; 4 = pseudoextinct order is polyphyletic or paraphyletic (only applies to nine orders with two or more taxa); 5 = pseudoextinct order is monophyletic but with different intraordinal relationships (only applies to five orders with three or more taxa). Abbreviations: HPAs, hypothetical predicted ancestors.

Pseudoextinction analyses with the molecular scaffold by itself recovered the highest number of splits on the MSST and therefore had the lowest RF distances (mean = 5.58) (Table 1). However, mean RF distances were only slightly higher in other analyses that incorporated a molecular scaffold (scaffold + fossils = 5.79, scaffold + fossils + HPA = 6.21, scaffold + HPA = 6). The analyses that incorporated a molecular scaffold and HPAs had the lowest median RF distance (2) whereas the three other analyses that incorporated a molecular scaffold had the same median RF distance (4) (Table 1 and Figs 2–5). By contrast, mean/median RF distances were much higher for analyses that lacked a molecular scaffold (fossils +HPA = 27.49/30, fossils = 44.53/44, HPA = 36.16/34) (Table 1). Among the 19 placental orders that were treated as pseudoextinct, mean RF distances across seven different analyses were lowest for Sirenia (14.14), Dermoptera (14.86), Lagomorpha (14.86), Primates (15.14), and Scandentia (15.57). Median RF distances across seven different analyses were lowest for Proboscidea (0), Sirenia (1), Cingulata (2), Dermoptera (2), Lagomorpha (2), Pilosa (2), Primates (2) and Scandentia (2). Pseudoextinct placental orders with the highest mean RF distances, in turn, were Afrosoricida (25.43), Chiroptera (25.14), Eulipotyphla (24.43), Pholidota (23.43), Cetartiodactyla (22.86) and Tubulidentata (21.9), and pseudoextinct placental orders with the highest median RF distances were Cetartiodactyla (24), Eulipotyphla (19), Afrosoricida (18), Carnivora (16), Chiroptera (16), Pholidota (14), and Tubulidentata (14).

Table 2 summarizes the extent to which pseudoextinct and other taxa were recovered in positions that violate the molecular scaffold. These misplacements are limited to pseudoextinct taxa in analyses that incorporated a scaffold, but can potentially affect any taxon in analyses that did not employ a scaffold. In analyses that incorporated a scaffold, 11 to 12 of the 19 placental orders (58–63%) were recovered in an incorrect position. Further, in five to eight instances (26–42%) the correct superordinal group (Afrotheria, Euarchontoglires, Laurasiatheria, Xenarthra) for the pseudoextinct order was not recovered as monophyletic. One order (5%) was recovered as polyphyletic or paraphyletic in analyses with the molecular scaffold and HPAs. Three orders (16%) were recovered as polyphyletic or paraphyletic in analyses with the molecular scaffold by itself, and this increased to four orders (21%) in analyses with the scaffold and fossils (Table 2). However, the combination of scaffold + HPAs + fossils resulted in zero pseudoextinct orders that were recovered as polyphyletic or paraphyletic (Table 2). One to three pseudoextinct orders (5–16%) were recovered as monophyletic but with intraordinal relationships that conflicted with the scaffold in analyses that incorporated a scaffold for the non-pseudoextinct orders (Table 2). In the three analyses that did not incorporate a molecular scaffold, placental orders were recovered in an incorrect position in 12 to 15 cases (63–79%), at least one superordinal group was not recovered as monophyletic in 13 to 19 cases (68–100%), zero to three orders (0–16%) were recovered as polyphyletic or paraphyletic, and two to three of the orders (11–16%) with three or more taxa exhibited altered intraordinal relationships that are incongruent with the scaffold.

Among individual placental orders, ten orders (53%) were always estimated in an incorrect interordinal position that violates the molecular scaffold. In five of these cases (Afrosoricida, Chiroptera, Eulipotyphla, Pholidota, Tubulidentata) the pseudoextinct order was misplaced outside of its correct superordinal group. By contrast, only two orders (11%) (Proboscidea, Sirenia) were always estimated with the correct sister-group relationship. However, even in these cases the larger superordinal group (Afrotheria) containing these orders was not always recovered. Three orders (16%) (Afrosoricida, Cetartiodactyla, Eulipotyphla) were estimated as polyphyletic or paraphyletic in at least three of the analyses. Notably, the inclusion of HPAs increased the monophyly of pseudoextinct orders. Indeed, two of the four analyses with HPAs (scaffold + fossils + HPAs, fossils + HPAs) yielded monophyly for every pseudoextinct order. Intraordinal relationships for monophyletic orders were always incorrectly estimated when Chiroptera was pseudoextinct. Specifically, the Old World fruitbat (*Pteropus giganteus*) was always estimated as the sister group to all of the other bats rather than as the sister group to the other yinpterochiropteran (*Rhinopoma hardwickii*). Other orders with incorrect intraordinal relationships when the order was monophyletic included Rodentia (five analyses), Cetartiodactyla (three analyses), and Eulipotyphla (two analyses).

## Discussion

### The accuracy of morphological phylogenetics

The accurate placement of extinct taxa in the Tree of Life is a fundamental challenge in phylogenetics. Because the placement of most extinct taxa cannot be vetted with molecular data, the accuracy of morphological phylogenetics must be assessed with other approaches such as pseudoextinction analyses that are performed upon extant taxa. Previous pseudoextinction analyses using O’Leary et al.’s [31] morphological data set suggest that morphology alone is insufficient for accurate phylogenetic reconstructions of the majority of pseudoextinct orders [15]. Recently, Beck and Baillie [32] employed a molecular scaffold from Meredith et al. [33] and reconstructed hypothetical predicted ancestors for morphological characters in O’Leary et al.’s [31] data set. These authors concluded that the inclusion of HPAs and fossils in a phylogenetic analysis was sufficient to accurately place all 19 placental orders into their correct superordinal group (Xenarthra, Afrotheria, Laurasiatheria, Euarchontoglires) using morphological data alone. Asher et al. [34] obtained similar results for Glires and found that the inclusion of HPAs nearly tripled the number of well-corroborated groups that were recovered in their morphological phylogenetic analysis of this clade. Importantly, both of these studies estimated and included the immediate HPAs of all extant taxa in their morphological phylogenetic analyses [32, 34]. However, the immediate HPAs of most extinct taxa cannot be reconstructed in scaffold-based analyses because molecular data cannot be procured for these taxa. In this study, we combined HPAs with pseudoextinction analyses to determine if HPAs elsewhere in the tree are sufficient to determine the accurate placement of each ‘extinct’ placental order when its immediate HPA is unknown. We also compared the general performance of molecular scaffolds, fossil taxa, HPAs, and combinations thereof for determining the accurate placement of pseudoextinct taxa.

Our results corroborate the results of Springer et al. [15] and show morphological characters by themselves cannot be relied upon to accurately place placental orders when they are pseudoextinct. Indeed, all seven sets of analyses failed to place the majority of 19 pseudoextinct placental orders next to their correct sister group. Analyses with the molecular scaffold (11 to 12 or 58–63% incorrect sister groups) performed slightly better than analyses that excluded the molecular scaffold (12 to 15 or 63–79% incorrect sister groups). Moreover, at least one of the four superordinal groups was not recovered as monophyletic in 11 to 17 cases (58–89%) when placental orders were treated as pseudoextinct (Table 2). These results stand in sharp contrast to the analysis of Asher et al. [34] that employed HPAs but no pseudoextinction. If pseudoextinct orders are a proxy for extinct orders, the inclusion of HPAs will not result in the accurate placement of pseudoextinct orders when the immediate hypothetical ancestor is unknown. Nevertheless, the inclusion of HPAs, with or without fossils, resulted in greater congruence with well-established clades than analyses with extant taxa only [15] or with the inclusion of fossils but not HPAs (also see [34]).

### The challenge of placing insectivores and myrmecophages

Ten placental orders (53%) (Afrosoricida, Carnivora, Chiroptera, Dermoptera, Eulipotyphla, Macroscelidea, Pholidota, Primates, Scandentia, Tubulidentata) were recovered in incorrect phylogenetic positions in all seven analyses. Five of these orders (Afrosoricida, Dermoptera, Eulipotyphla, Macroscelidea, Scandentia) are comprised of taxa that Wagner [50] included in his Insectivora. The polyphyletic origins of Wagner’s Insectivora were recognized by morphologists who removed menotyphlan insectivores from this group and placed them in the orders Dermoptera, Macroscelidea, and Scandentia [51, 52]. However, morphologists continued to support the monophyly of Lipotyphla (solenodons, moles, shrews, hedgehogs, tenrecs, golden moles) [51, 53–55] until this group was shown to be polyphyletic based on molecular data and divided into Afrosoricida and Eulipotyphla [56–59]. Even with the general acceptance of lipotyphlan polyphyly, cladistic analyses of morphology-only data sets have continued to support the monophyly of this clade [31]. Our analyses confirm the difficulty of correctly placing “insectivores” in cladistic analyses with morphological data. Indeed, Afrosoricida and Eulipotyphla always join each other when one of these groups is pseudoextinct.

Beyond the various “insectivore” groups (Insectivora, Menotyphla, Lipotyphla) that have subsequently been shown to be polyphyletic, Edentata is a polyphyletic taxon that is historically comprised of the myrmecophagous orders Xenarthra, Pholidota [22–24] and sometimes Tubulidentata [31]. Two of these orders (Pholidota, Tubulidentata) are among those that were always misplaced in our pseudoextinction analyses. Pholidotans were always the sister taxon to Xenarthra or Pilosa when pseudoextinct. Tubulidentata, in turn, were usually the sister taxon to other myrmecophagous taxa (i.e., Pholidota, Xenarthra, Pholidota + Xenarthra) although in two cases (Scaffold, Scaffold + HPAs) were the sister taxon to Euungulata (Cetartiodactyla + Perissodactyla). With the exception of Tubulidentata + Euungulata, these results represent different incarnations of Edentata and underscore the challenges that are associated with accurately placing myrmecophagous taxa in morphological phylogenetic analyses. The order Carnivora is represented by *Canis familiaris* (dog) and *Felis silvestris* (European wildcat) in O’Leary et al.’s [29] data set. With a few exceptions (e.g., *Proteles cristata*), the members of Carnivora are not insectivorous/myrmecophagous. However, the sister taxon to Carnivora is an edentulous, myrmecophagous order (Pholidota) [33] and was misplaced in all seven analyses when pseudoextinct. This result further highlights the difficulty of reconstructing clades such as Ferae (Carnivora + Pholidota) that include myrmecophagous orders.

By contrast with our results for Pholidota and Tubulidentata, the myrmecophagous *Tamandua tetradactyla* (Order Pilosa) and largely myrmecophagous *Dasypus novemcinctus* (Order Cingulata) were often recovered as sister groups to each other when pseudoextinct (Cingulata in 6 of 7 analyses; Pilosa in 5 of 7 analyses; see Table 2). Previously Springer et al. [15] performed a pseudoextinction analysis with O’Leary et al.’s [31] data set and treated Cingulata and Pilosa as a single order (Xenarthra). Pseudoextinction analyses with Xenarthra resulted in this taxon jumping inside of Afrotheria and joining with Tubulidentata [15]. Similarly, Springer et al. [12] performed pseudoextinction analyses with Xenarthra and a much smaller data set (185 osteological characters; [60]), and recovered Cingulata as sister to Tubulidentata and Pilosa as sister to Pholidota. These results suggest that morphological data will recover more accurate positions for extinct taxa when a suitably close living relative is also included in the analysis.

### Effect of HPAs

The inclusion of the HPAs resulted in a greater number of pseudoextinct orders that were recovered as monophyletic. For two analyses, Scaffold + Fossils + HPA and Fossils + HPA, all pseudoextinct orders were recovered as monophyletic. In the Scaffold + HPA and HPA only analyses only one pseudoextinct order (5%) (Cetartiodactyla) was not monophyletic. In the HPA only analyses Cetacea was recovered outside of an ungulate clade that included Perissodactyla and terrestrial Cetartiodactyla. In the Scaffold + HPA analyses, Cetacea was recovered as the sister group to Sirenia. The increase in the number of monophyletic (pseudoextinct) orders that resulted from the inclusion of HPAs suggests that increased fossil sampling may also increase the accuracy of morphological phylogenetic analyses if a sufficient number of transitional fossils with appropriate combinations of primitive and derived characters are included in the analysis.

However, HPAs may not replicate extinct taxa in the fossil record [34]. Thus, new fossil discoveries may not have the same positive effect as HPAs when they are included in a cladistic analysis. Additionally, the fossil record is highly incomplete and many gaps in the fossil record will likely be permanent due to environmental factors that did not favor fossilization [61]. Thus, even with additional fossil discoveries, it is unlikely that all ancestors for a large group will ever be known. Our results show that the absence of even a single ancestor can be severely detrimental to the accurate phylogenetic reconstruction of some pseudoextinct groups. We acknowledge that the analyses of Beck and Baillie [32] represent a best-case scenario for future fossil discoveries. It remains to be tested if close relatives that are not ancestral would be as beneficial as ancestral species to improve the performance of morphological phylogenetic analyses.

## Future directions

Fossil species comprise the majority of taxa in the mammalian Tree of Life. The discovery of fossil species and the accurate placement of these fossils in phylogenetic trees is crucial for both molecular dating analyses and reconstructing temporal sequences of character acquisition. The inclusion of hypothetical predicted ancestors is not a panacea for morphological phylogenetic analyses with extinct taxa, but the inclusion of HPAs may increase the accuracy of morphological phylogenetic analyses relative to analyses that lack these ancestors. It is crucial to continue using fossil taxa to elucidate the evolutionary history of eutherian mammals. Despite our results, it is possible that additional fossil taxa will increase the accuracy of morphological phylogenetics. O’Leary et al.’s [31] data set includes far more morphological characters (>4500) than any other data set for mammals, but only includes 40 fossil taxa that span a vast amount of evolutionary time (from ~200 million years ago to the Recent). The inclusion of additional fossil taxa may break up long branches that are prone to long-branch attraction and improve the accuracy of morphological phylogenetics in estimating the higher-level phylogeny of mammals. Future work should aim to increase the diversity of fossil taxa that are used alongside extant taxa in morphological or total-evidence analyses.

## Supporting information

S1 Fig Fossil taxa + hypothetical predicted ancestors analyses. All pruned trees for one of our seven sets of analyses (fossil taxa + hypothetical predicted ancestors) including the trees that resulted from an analysis where no taxa were pseudoextinct. In agreement with Beck and Baillie [32], both most parsimonious trees (MPTs) that were recovered when no taxa were pseudoextinct reconstructed all four placental superorders (Afrotheria, Xenarthra, Laurasiatheria, Euarchontoglires) as monophyletic. All pseudoextinction analyses recovered a single MPT except for Carnivora (2 MPTs), Eulipotyphla (2 MPTs), Macroscelidea (2 MPTs), Scandentia (2 MPTs) and Tubulidentata (3 MPTs).
(PDF)

S2 Fig Fossil taxa analyses. All pruned trees for one of our seven sets of analyses (fossil taxa only) including the tree that resulted from an analysis where no taxa were pseudoextinct. The most parsimonious tree (MPT) found when no taxa were pseudoextinct failed to recover Afrotheria, Laurasiatheria, and Euarchontoglires as monophyletic. Xenarthra, while monophyletic, was recovered within a larger “edentate” group including Pholidota and Tubulidentata. All pseudoextinction analyses found a single MPT except for Carnivora and Hyracoidea, which each had two MPTs.
(PDF)

S3 Fig Hypothetical predicted ancestors analyses. All pruned trees for one of our seven sets of analyses (hypothetical predicted ancestors only) including the trees that resulted from an analysis where no taxa were pseudoextinct. The most parsimonious trees (MPTs) found when no taxa were pseudoextinct failed to recover Afrotheria, Xenarthra, and Laurasiatheria as monophyletic. Euarchontoglires was recovered as monophyletic in all 3 MPTs that lacked pseudoextinct taxa, although in each case the interordinal relationships within Euarchontoglires and intraordinal relationships within Rodentia conflicted with the molecular scaffold. All pseudoextinction analyses found a single MPT exception for Cetartiodacyla (2 MPTs), Proboscidea (3 MPTs), Rodentia (2 MPTs), Sirenia (3 MPTs), and Tubulidentata (2 MPTs).
(PDF)
