# Supplementary material for: The effects of fossil taxa, hypothetical predicted ancestors, and a molecular scaffold on pseudoextinction analyses of extant placental orders
Source: PLoS One. 2021 Sep 17;16(9):e0257338. doi: 10.1371/journal.pone.0257338 (PMC8448315; doi:10.1371/journal.pone.0257338)

1 ("MSST")

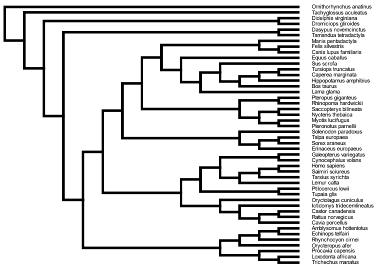

2 ("NoPseudoextinct")

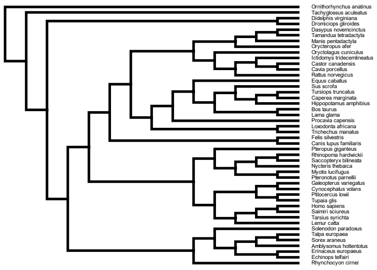

3 ("Afroscoricida")

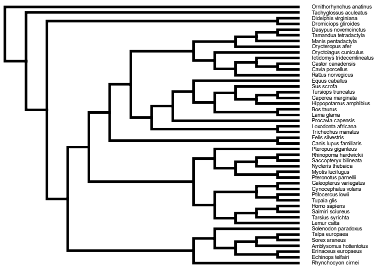

4 ("Carnivora1")

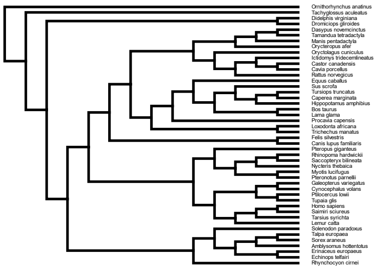

5 ("Carnivora2")

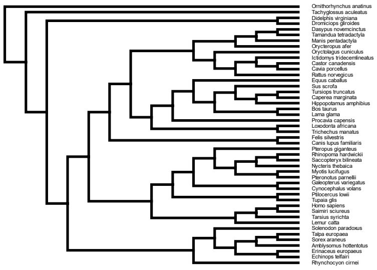

6 ("Cetartiodactyla")

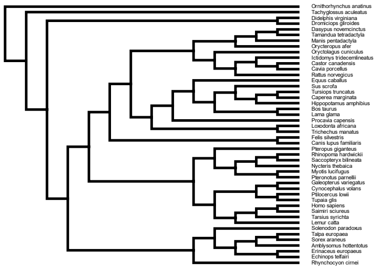

7 ("Chiroptera")

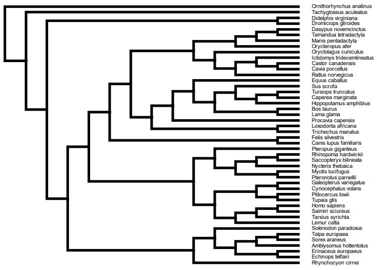

8 ("Cingulata")

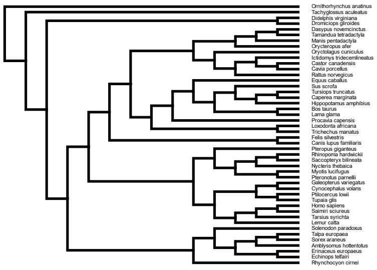

9 ("Dermoptera")

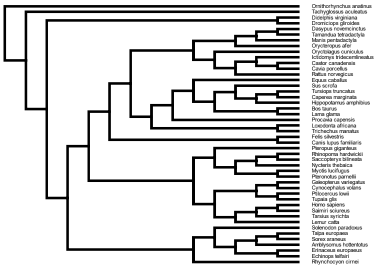

10 ("Eulipotyphla")

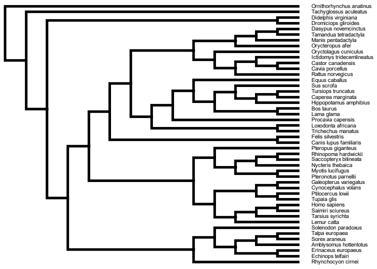

11 ("Hyracoidea1")

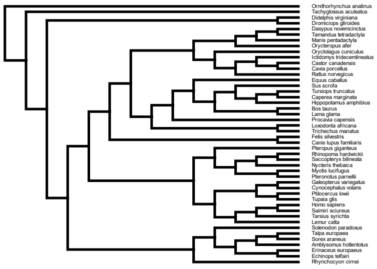

12 ("Hyracoidea2")

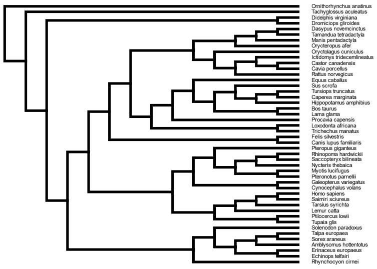

13 ("Lagomorpha")

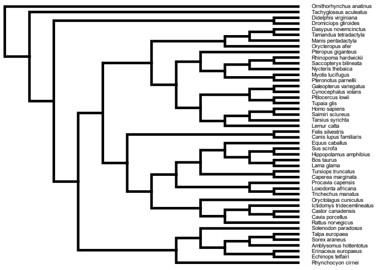

14 ("Macroscelidea")

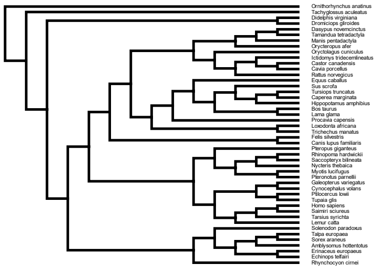

15 ("Perissodactyla")

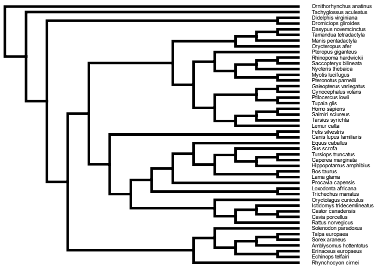

16 ("Pholidota")

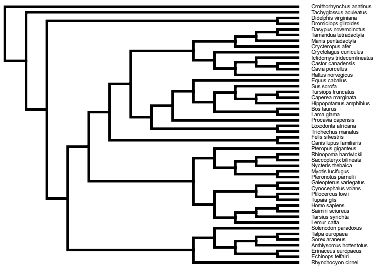

17 ("Pilosa")

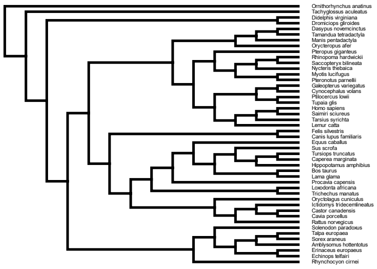

18 ("Primates")

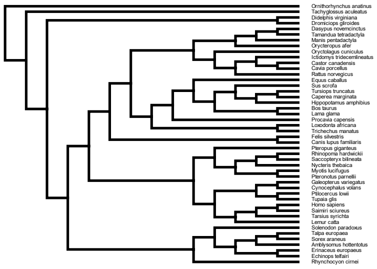

19 ("Proboscidea")

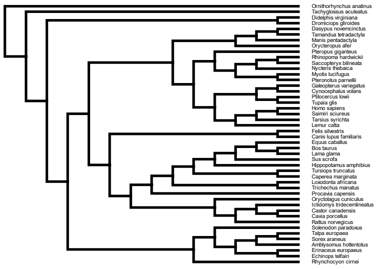

20 ("Rodentia")

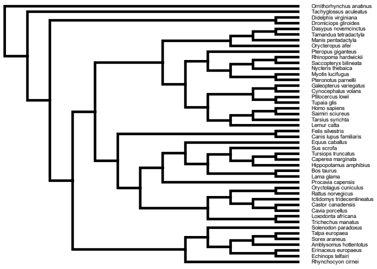

21 ("Scandentia")

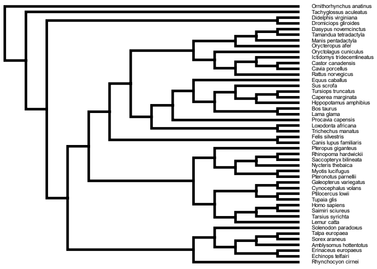

22 ("Sirenia")

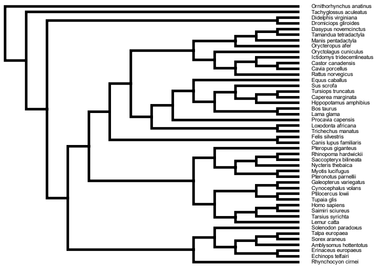

23 ("Tubulidentata")

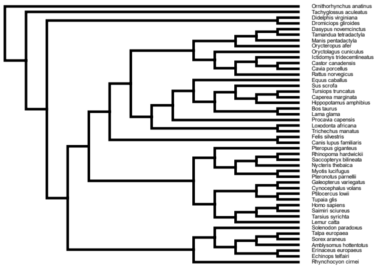

Supplement: S2 Fig — All pruned trees for one of our seven sets of analyses (fossil taxa only) including the tree that resulted from an analysis where no taxa were pseudoextinct. The most parsimonious tree (MPT) found when no taxa were pseudoextinct failed to recover Afrotheria, Laurasiatheria, and Euarchontoglires as monophyletic. Xenarthra, while monophyletic, was recovered within a larger “edentate” group including Pholidota and Tubulidentata. All pseudoextinction analyses found a single MPT except for Carnivora and Hyracoidea, which each had two MPTs. (PDF) [file pone.0257338.s002.pdf]
