# Supplementary material for: The effects of fossil taxa, hypothetical predicted ancestors, and a molecular scaffold on pseudoextinction analyses of extant placental orders
Source: PLoS One. 2021 Sep 17;16(9):e0257338. doi: 10.1371/journal.pone.0257338 (PMC8448315; doi:10.1371/journal.pone.0257338)

1 ("MSST")

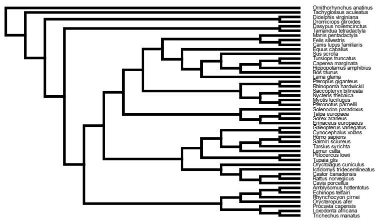

2 ("NoPseudoextinct 1")

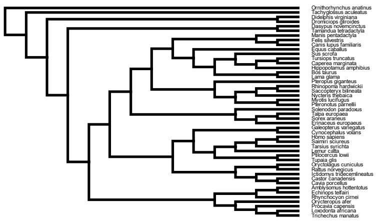

3 ("NoPseudoextinct 2")

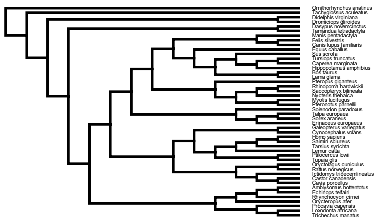

4 ("Afroscoricida")

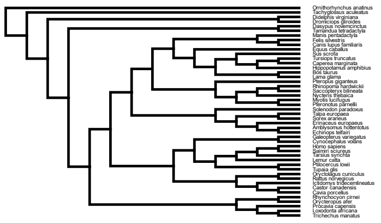

5 ("Carnivora 1")

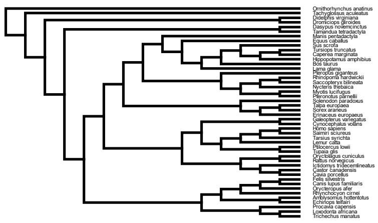

6 ("Carnivora 2")

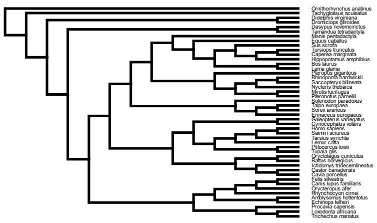

7 ("Cetartiodactyla")

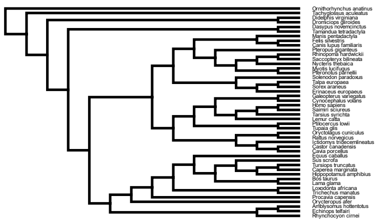

8 ("Chiroptera")

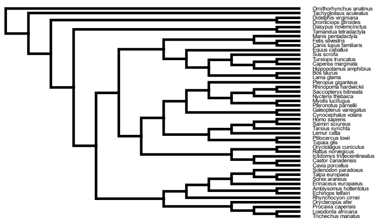

9 ("Cingulata")

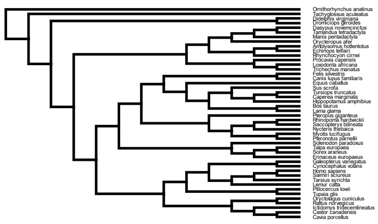

10 ("Dermoptera")

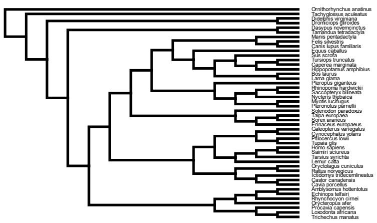

11 ("Eulipotyphla 1")

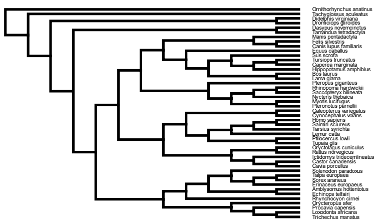

12 ("Eulipotyphla 2")

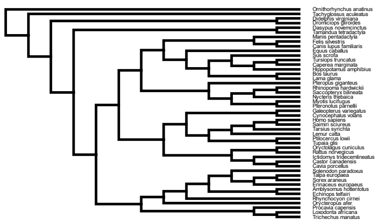

13 ("Hyracoidea")

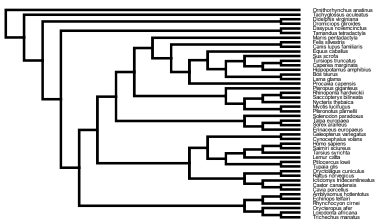

14 ("Lagomorpha")

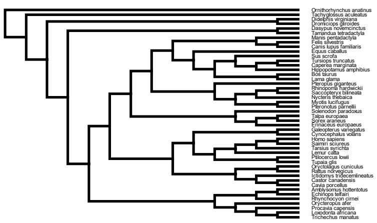

15 ("Macroscelidea 1")

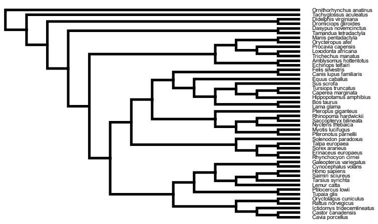

16 ("Macroscelidea 2")

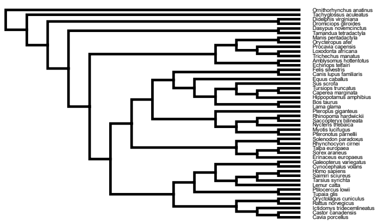

17 ("Perissodactyla")

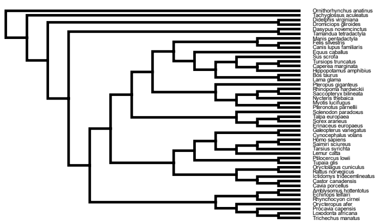

18 ("Pholidota")

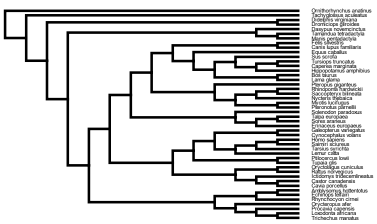

19 ("Pilosa")

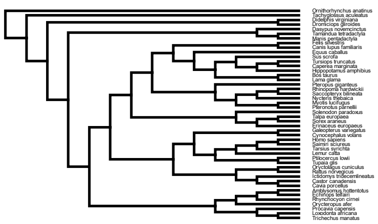

20 ("Primates")

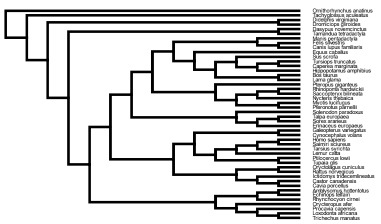

21 ("Proboscidea")

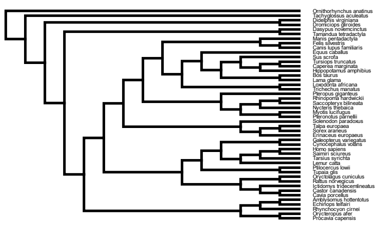

22 ("Rodentia")

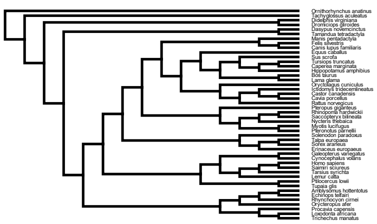

23 ("Scandentia 1")

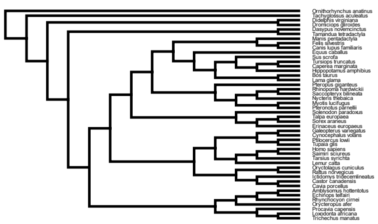

24 ("Scandentia 2")

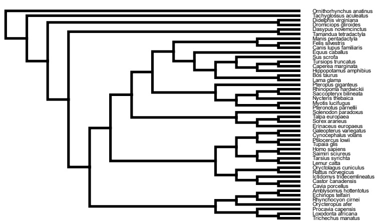

25 ("Sirenia")

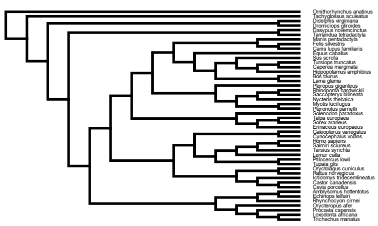

26 ("Tubulidentata 1")

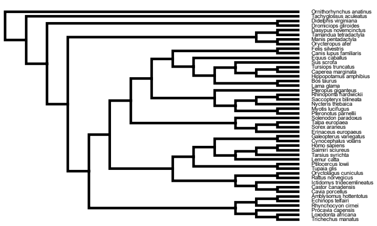

27 ("Tubulidentata 2")

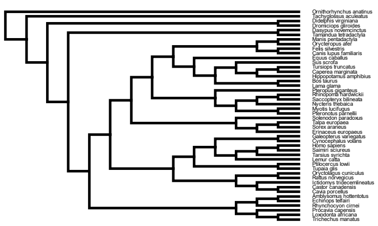

28 ("Tubulidentata 3")

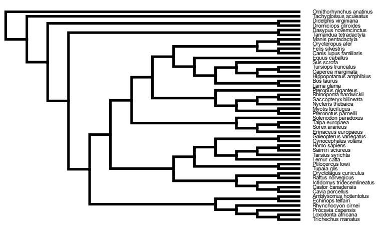

Supplement: S1 Fig — All pruned trees for one of our seven sets of analyses (fossil taxa + hypothetical predicted ancestors) including the trees that resulted from an analysis where no taxa were pseudoextinct. In agreement with Beck and Baillie [32], both most parsimonious trees (MPTs) that were recovered when no taxa were pseudoextinct reconstructed all four placental superorders (Afrotheria, Xenarthra, Laurasiatheria, Euarchontoglires) as monophyletic. All pseudoextinction analyses recovered a single MPT except for Carnivora (2 MPTs), Eulipotyphla (2 MPTs), Macroscelidea (2 MPTs), Scandentia (2 MPTs) and Tubulidentata (3 MPTs). (PDF) [file pone.0257338.s001.pdf]
