# Supplementary material for: The effects of fossil taxa, hypothetical predicted ancestors, and a molecular scaffold on pseudoextinction analyses of extant placental orders
Source: PLoS One. 2021 Sep 17;16(9):e0257338. doi: 10.1371/journal.pone.0257338 (PMC8448315; doi:10.1371/journal.pone.0257338)

1 ("MSST")

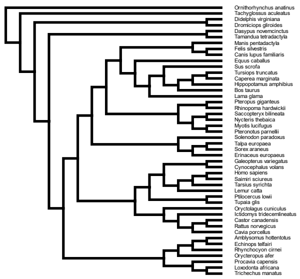

2 ("NoPseudoextinct1")

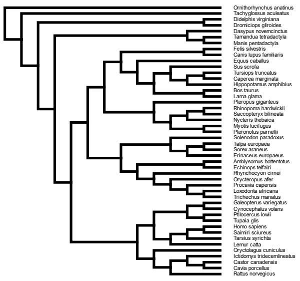

3 ("NoPseudoextinct2")

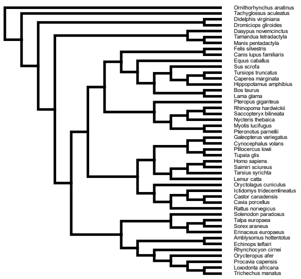

4 ("NoPseudoextinct3")

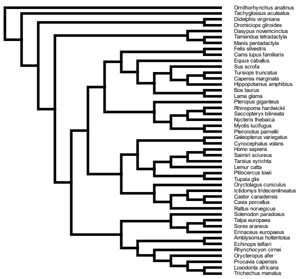

5 ("Afroscoricida")

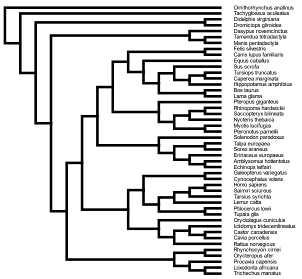

6 ("Carnivora")

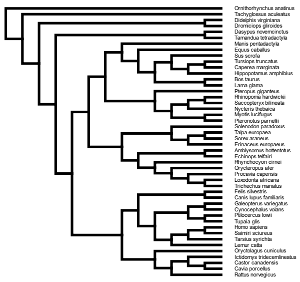

7 ("Cetartiodactyla1")

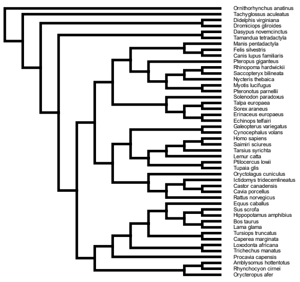

8 ("Cetartiodactyla2")

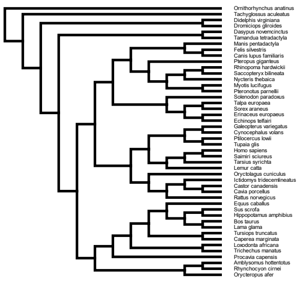

9 ("Chiroptera")

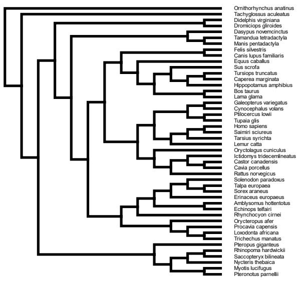

10 ("Cingulata")

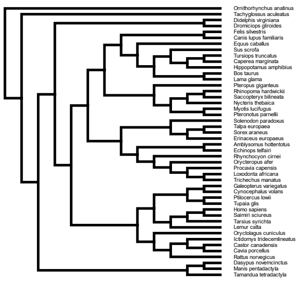

11 ("Dermoptera")

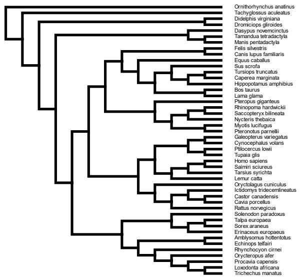

12 ("Eulipotyphla")

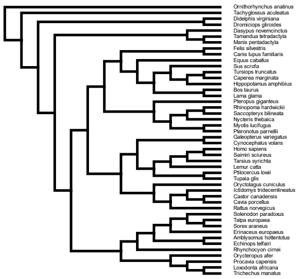

13 ("Hyracoidea")

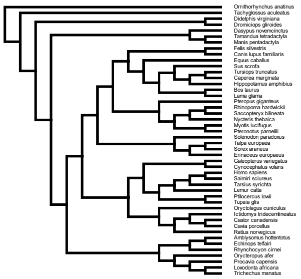

14 ("Lagomorpha")

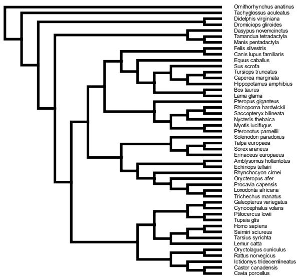

15 ("Macroscelidea")

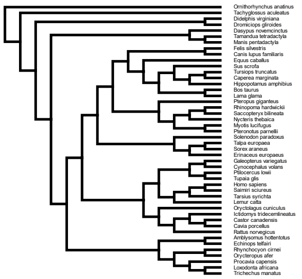

16 ("Perissodactyla")

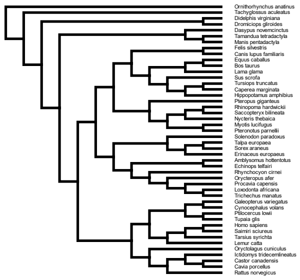

17 ("Pholidota")

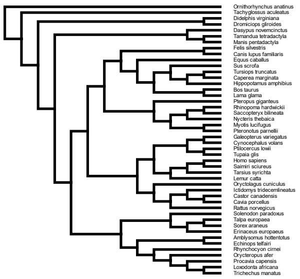

18 ("Pilosa")

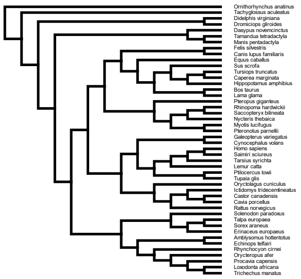

19 ("Primates")

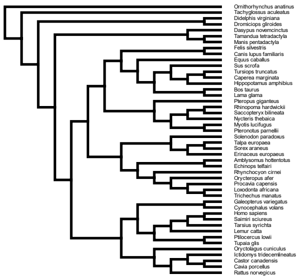

20 ("Proboscidea1")

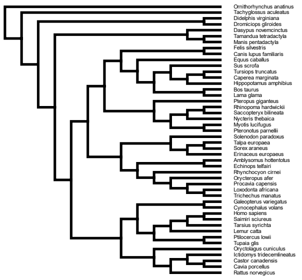

21 ("Proboscidea2")

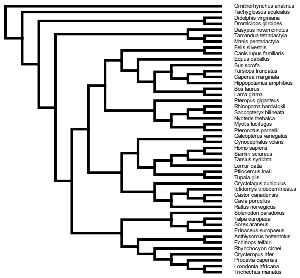

22 ("Proboscidea3")

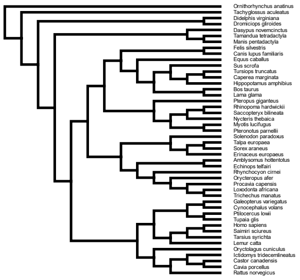

23 ("Rodentia1")

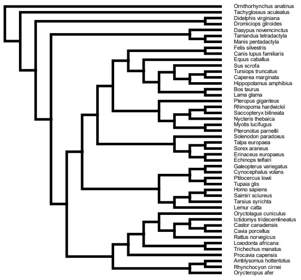

24 ("Rodentia2")

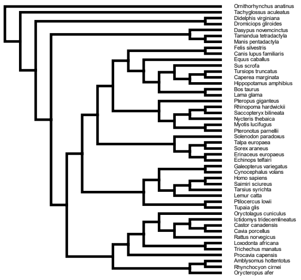

25 ("Scandentia")

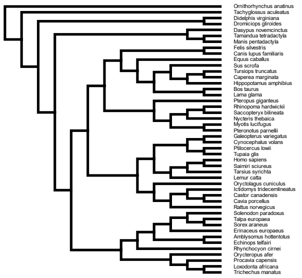

26 ("Sirenia1")

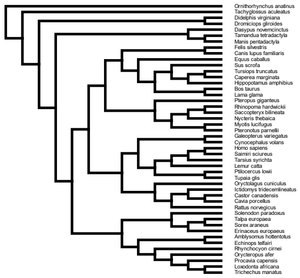

27 ("Sirenia2")

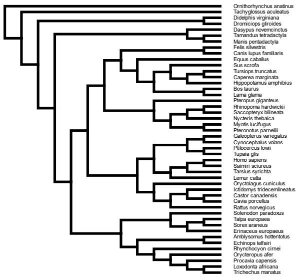

28 ("Sirenia3")

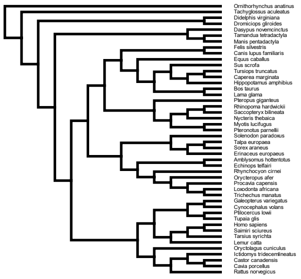

29 ("Tubulidentata1")

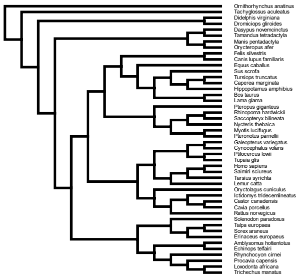

30 ("Tubulidentata2")

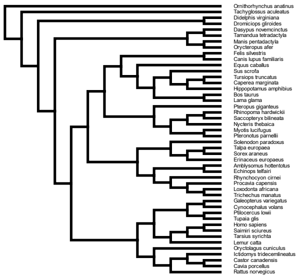

Supplement: S3 Fig — All pruned trees for one of our seven sets of analyses (hypothetical predicted ancestors only) including the trees that resulted from an analysis where no taxa were pseudoextinct. The most parsimonious trees (MPTs) found when no taxa were pseudoextinct failed to recover Afrotheria, Xenarthra, and Laurasiatheria as monophyletic. Euarchontoglires was recovered as monophyletic in all 3 MPTs that lacked pseudoextinct taxa, although in each case the interordinal relationships within Euarchontoglires and intraordinal relationships within Rodentia conflicted with the molecular scaffold. All pseudoextinction analyses found a single MPT exception for Cetartiodacyla (2 MPTs), Proboscidea (3 MPTs), Rodentia (2 MPTs), Sirenia (3 MPTs), and Tubulidentata (2 MPTs). (PDF) [file pone.0257338.s003.pdf]
